# Supplementary material for: Protection and consolidation of stone heritage by self-inoculation with indigenous carbonatogenic bacterial communities
Source: Nat Commun. 2017 Aug 17;8:279. doi: 10.1038/s41467-017-00372-3 (PMC5561188; doi:10.1038/s41467-017-00372-3)
Supplement: Supplementary file 1 — Supplementary Information [file 41467_2017_372_MOESM1_ESM.pdf]

File name: Supplementary Information
Description: Supplementary figures, supplementary table and supplementary references.

File name: Peer review file
Description:

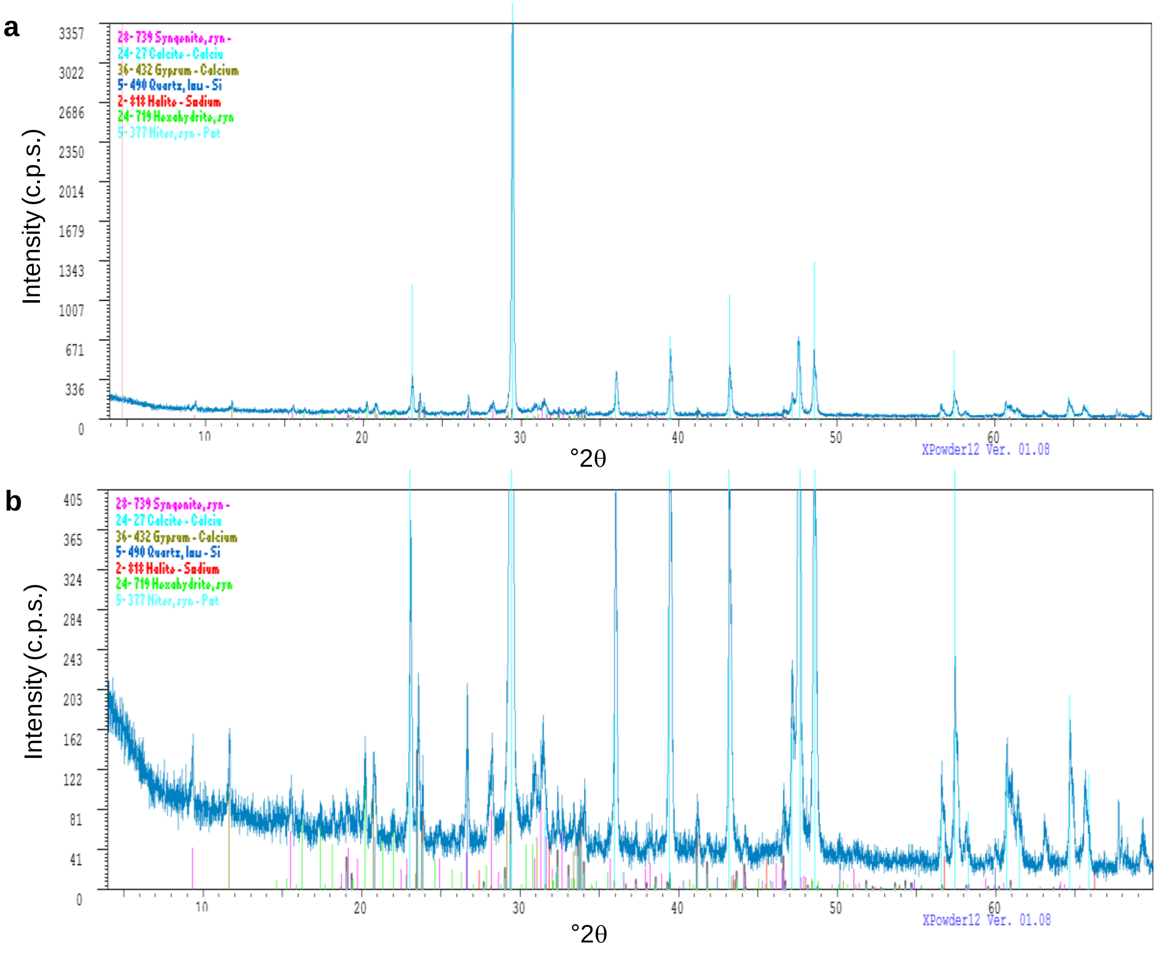

Supplementary Fig. 1. **XRD pattern of calcarenite stone samples collected from San Jeronimo Monastery**. a) Full intensity diffractogram, and b) enlarged view of the less intense Bragg peaks in (a). In addition to calcite (main phase) minor phases identified include: quartz (1±0.6 wt%) and salts including syngenite (4.5±0.6 wt%), niter (4.2±0.5 wt%), hexahydrite (2.7±0.6 wt%) gypsum (2.3±0.7 wt%) and halite (0.2±0.1 wt%).

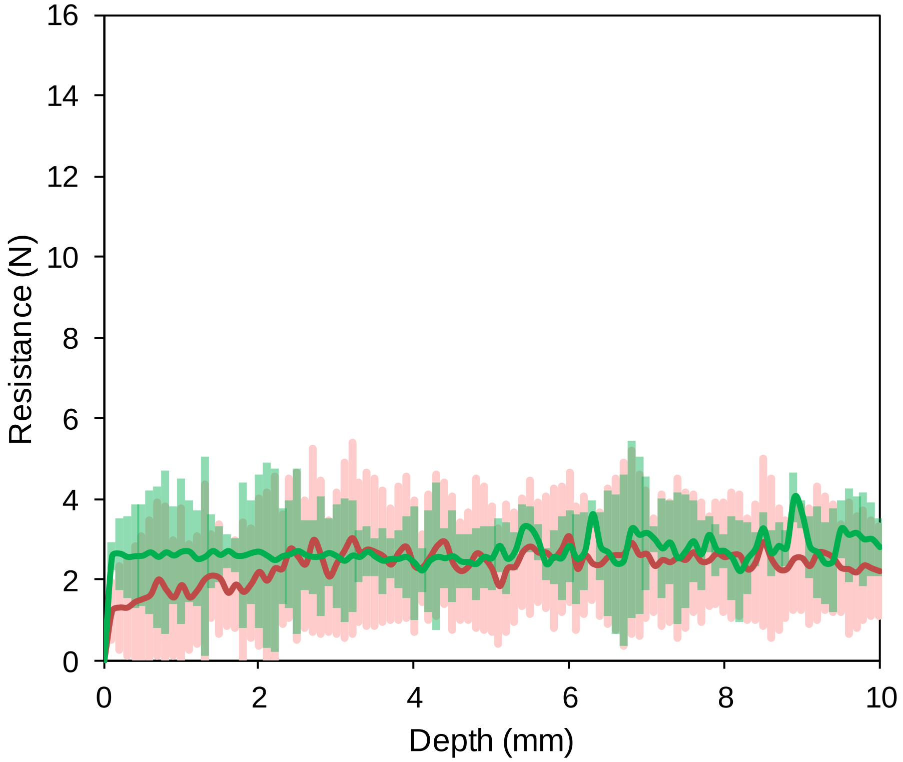

Supplementary Fig. 2. Drilling resistance (DR) values of untreated calcarenite stone blocks at San Jeronimo Monastery. Average DR values and standard deviation for weathered (red curve) and unweathered (green curve) calcarenite stone blocks.

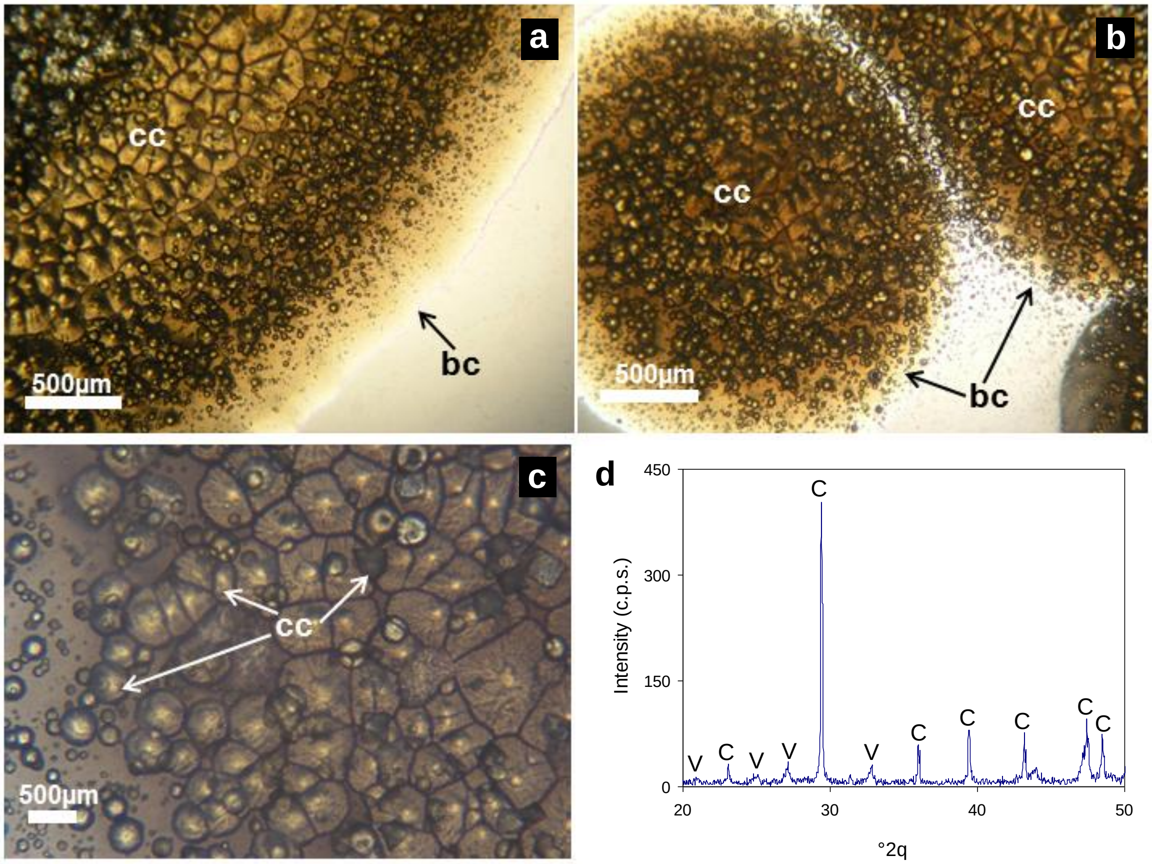

Supplementary Fig. 3. **Carbonatogenic capacity of the isolates obtained from the bacterial community**. a-c) Optical microscopy images of bacterial isolates obtained in M-3P solid medium showing parts of bacterial colonies (bc) and $CaCO_3$ precipitates (cc); d) XRD pattern of $CaCO_3$ precipitates showing the presence of calcite (C) and (minor) vaterite (V).

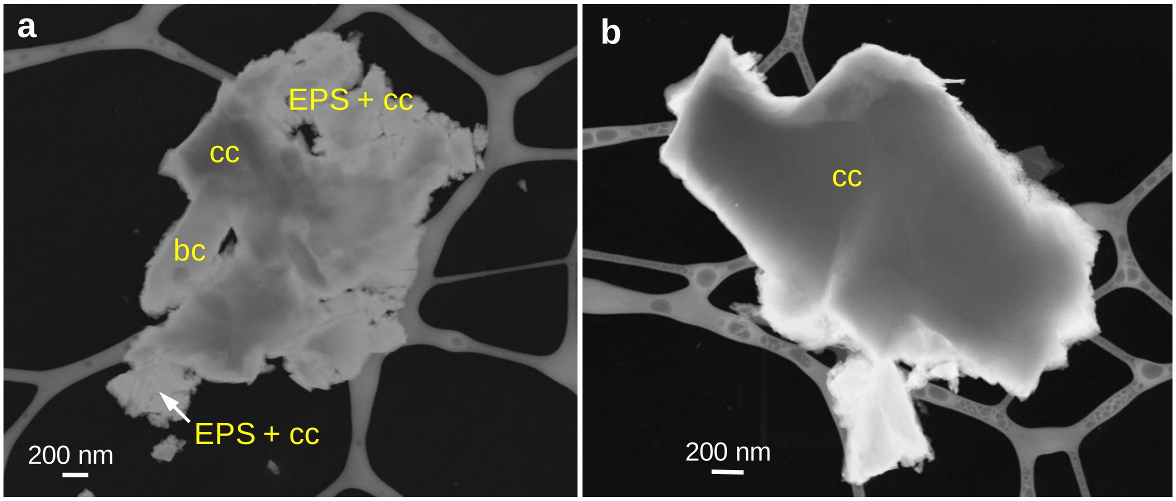

Supplementary Fig. 4. Negative STEM photomicrographs of bacterial calcite crystals. a) aggregate of calcite and organics formed after 24 h bacterial mineralization of $CaCO_3$. Organics (EPS) interspersed within nanogranular calcite (cc) and bacterial cells (bc) are observed; b) well developed bacterial calcite (cc) rhombohedral crystals after 48 h mineralization. Note that bacterial cells are no longer visible.

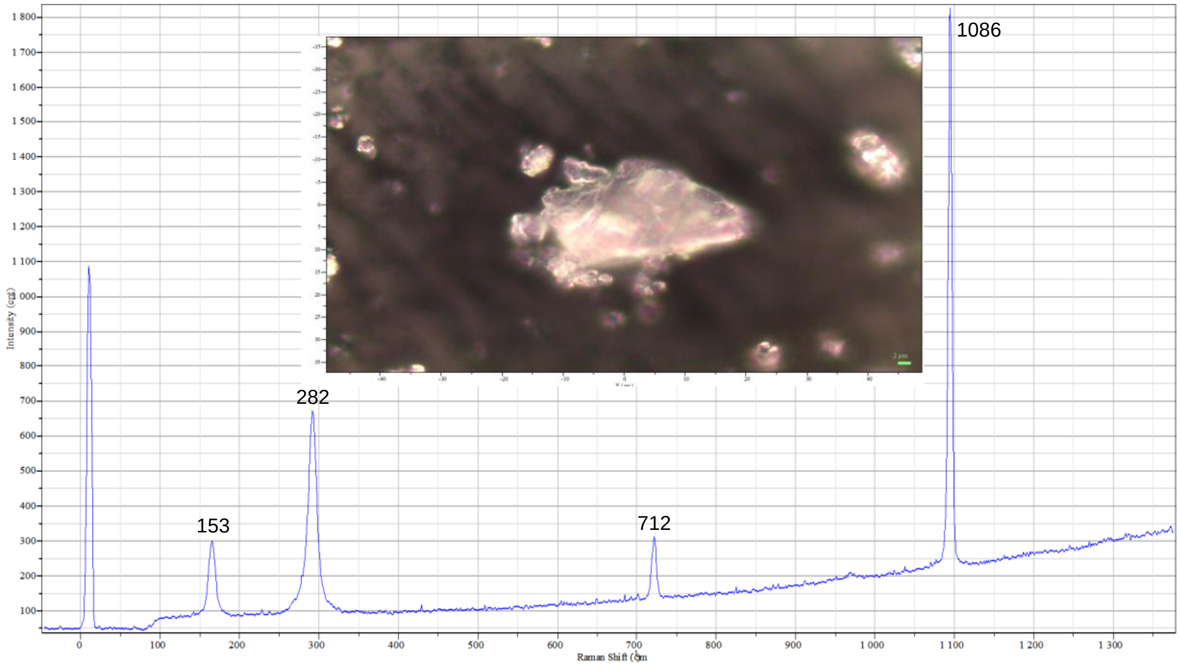

Supplementary Fig. 5. **Raman spectrum of bacterial calcite**. The main bands corresponding to calcite are observed. Optical microscopy image of analyzed bacterial calcite in inset.

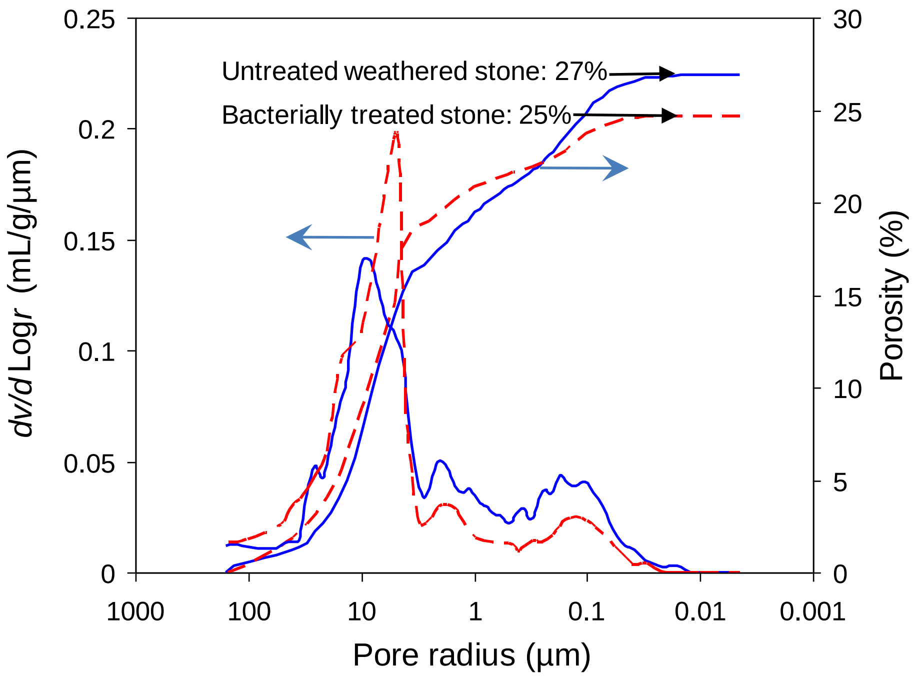

Supplementary Fig. 6. **Porosity and pore size distribution of calcarenite.** Representative MIP plots showing both the cumulative intrusion curves (i.e., porosity) and pore size distribution curves (i.e., log of differential intrusion, or d*v*/dLog*r*, versus *r*, where *v* is the intruded volume and *r* is the pore radius) for untreated (blue solid line) and biomineralized (red dashed line) stone blocks from San Jeronimo Monastery.

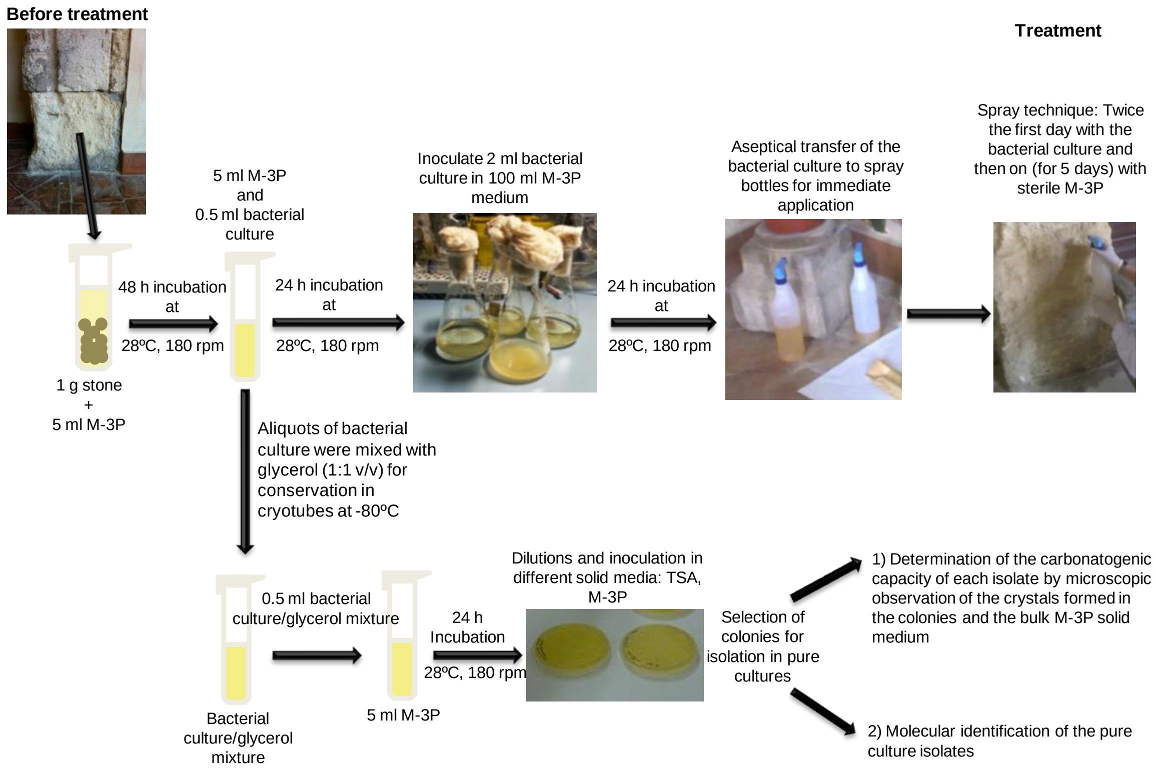

Supplementary Fig 7. Protocol for bacterial self-inoculation treatment. The scheme shows the protocol for the self-inoculation treatment involving the collection of bacteria from stone, their culture in the laboratory, and their subsequent in situ application (upper row) and the protocol for the isolation, determination of the carbonatogenic capacity, and molecular identification of pure culture isolates (lower row).

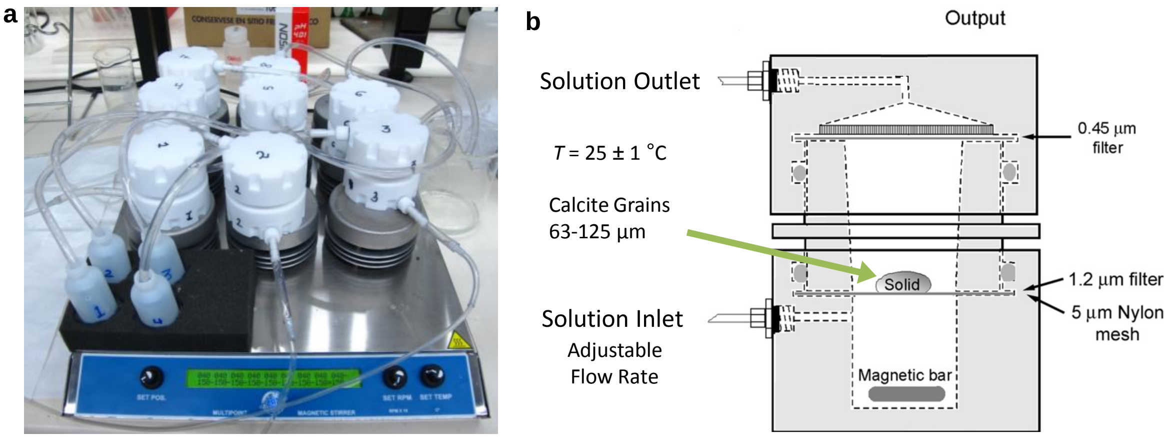

Supplementary Fig. 8. Experimental set up for macroscale dissolution tests. a) image of the Teflon reactors during experiment; b) scheme of a flow-through reactor (modified from ref. 1)

Supplementary Table 1. Color measurements in San Jeronimo Monastery before and 5, 12, and 24 months after application of the bio-consolidation treatments

		**Δ*L****	**Δ*a****	**Δ*b****	**Δ*E***	***p*-values**‡
**Control**†		75.2 ± 1.9	3.2 ± 0.5	19.2 ± 0.5	**2.1 ± 1.3**	-
**Treatment with the bacterial community (self-inoculation bio-treatment)**	At 5 months	73.2 ± 0.8	3.5 ± 0.4	20.4 ± 1.1	**2.4 ± 1.2**	0.924
	At 12 months	73.1 ± 0.6	3.4 ± 0.4	20.3 ± 0.6	**2.4 ± 1.3**	0.826
	At 24 months	71.9 ± 0.6	3.0 ± 0.2	17.4 ± 0.5	**3.8 ± 1.7**	0.015
**Treatment with *M. xanthus*+ M-3P**	At 5 months	71.8 ± 1.3	1.8 ± 0.3	19.1 ± 0.3	**3.6 ± 1.4**	0.056
	At 12 months	73.7 ± 0.9	2.0 ± 0.02	18.3 ± 0.8	**2.2 ± 1.2**	0.145
	At 24 months	70.9 ± 0.1	2.1 ± 0.2	18.5 ± 0.3	**4.5 ± 0.4**	0.051
**Treatment with M-3P nutritive solution**	At 5 months	72.7 ± 0.2	2.0 ± 0.02	19.2 ± 1.1	**2.6 ± 1.1**	0.203
	At 12 months	72.7 ± 2.5	1.9 ± 0.2	17.5 ± 0.3	**3.5 ± 2.5**	0.041
	At 24 months	75.3 ± 0.4	2.9 ± 0.1	20.3 ± 0.4	**1.1 ± 0.5**	0.788

† Average (± st. dev.) color variation among different untreated stone blocks/areas
‡ *p*-values from *t*-tests (representing statistical significance)

Supplementary References

1. Rozalen, M. L., Huertas, F. J., Brady, P. V., Cama, J., Garcia-Palma, S., & Linares, J. Experimental study of the effect of pH on the kinetics of montmorillonite dissolution at 25 degrees C. *Geochim. Cosmochim. Acta* **72**, 4224-4253 (2008).
